# Molecular targets for the protodynamic action of cis-urocanic acid in human bladder carcinoma cells

**DOI:** 10.1186/1471-2407-10-521

**Published:** 2010-10-03

**Authors:** Emilia Peuhu, Aura Kaunisto, Jarmo K Laihia, Lasse Leino, John E Eriksson

**Affiliations:** 1 Turku Centre for Biotechnology, University of Turku and Åbo Akademi University, BioCity, FI-20520 Turku, Finland; 2 Department of Biosciences, Åbo Akademi University, FI-20520 Turku, Finland; 3 BioCis Pharma Ltd., Itäinen Pitkäkatu 4 B, FI-20520 Turku, Finland

## Abstract

**Background:**

cis-urocanic acid (cis-UCA) is an endogenous amino acid metabolite capable of transporting protons from the mildly acidic extracellular medium into the cell cytosol. The resulting intracellular acidification suppresses many cellular activities. The current study was aimed at characterizing the molecular mechanisms underlying cis-UCA-mediated cytotoxicity in cultured cancer cells.

**Methods:**

5367 bladder carcinoma cells were left untreated or treated with cis-UCA. Cell death was assessed by measuring caspase-3 activity, mitochondrial membrane polarization, formation and release of cytoplasmic histone-associated DNA fragments, and cellular permeabilization. Cell viability and metabolic activity were monitored by colorimetric assays. Nuclear labelling was used to quantify the effects of cis-UCA on cell cycle. The activity of the ERK and JNK signalling pathways was studied by immunoblotting with specific antibodies. Phosphatase activity in cis-UCA-treated cells was determined by assay kits measuring absorbance resulting from the dephosphorylation of an artificial substrate. All statistical analyses were performed using the two-way Student's t-test (p < 0.05).

**Results:**

Here we report that treatment of the 5637 human bladder carcinoma cells with 2% cis-UCA induces both apoptotic and necrotic cell death. In addition, metabolic activity of the 5637 cells is rapidly impaired, and the cells arrest in cell cycle in response to cis-UCA. Importantly, we show that cis-UCA promotes the ERK and JNK signalling pathways by efficiently inhibiting the activity of serine/threonine and tyrosine phosphatases.

**Conclusions:**

Our studies elucidate how cis-UCA modulates several cellular processes, thereby inhibiting the proliferation and survival of bladder carcinoma cells. These anti-cancer effects make cis-UCA a potential candidate for the treatment of non-muscle invasive bladder carcinoma.

## Background

cis-urocanic acid [cis-UCA; 3-(1*H*-imidazol-4-yl)prop-2-enoic acid] is an endogenous metabolite of amino acid histidine found in the mammalian skin after exposure to ultraviolet radiation. cis-UCA is known to induce immunosuppression in animals [1,2]. We have recently discovered that cis-UCA is capable of transporting protons from the mildly acidic extracellular medium into the cytosol of cancer cells, thereby inhibiting the proliferation of several human tumour cell lines [3,4], and inducing apoptosis *in vitro *and in tumour xenografts *in vivo *[4]. Intracellular acidification is known to affect cell proliferation [5,6] and to promote apoptosis [7,8]. This new mode of action of cis-UCA, which we have named as the *protodynamic *action, constitutes a novel concept of anticancer therapy. The transportation and release of protons into the cytosol is based on the unique second acid dissociation constant (pKa_2_) of cis-UCA [9], and it is inherently independent of enzymes and membrane receptors.

The use of cis-UCA as an anticancer agent against non-muscle-invasive bladder cancer has previously been evaluated in cultured moderately and poorly differentiated human transitional bladder cancer cells using a short term (up to 2 h) pulse treatment model *in vitro*, which mimics the clinical intravesical chemotherapy regimen [3]. In the current study, we aimed at characterizing the molecular mechanisms underlying cis-UCA-mediated cytotoxicity to cultured cancer cells. Here we show that cis-UCA extends a wide range of effects on bladder carcinoma cells. Treatment of 5637 bladder carcinoma cells with 2% cis-UCA induced a combination of apoptotic and necrotic cell death. In addition, metabolic activity of the 5637 cells was rapidly impaired, and cell cycle arrest was induced at 1-3% cis-UCA. Importantly, we were able to show that cis-UCA promotes the ERK and JNK signalling pathways. Rather than due to increased kinase activation, the observed effect seemed to be caused by a decrease in both tyrosine and serine/threonine phosphatase activities that act as critical regulators of the ERK and JNK signalling pathways. Within the scope of the protodynamic therapy concept, our study suggests a novel mechanism for cis-UCA-induced inhibition of cancer cell survival, based upon compromised signalling and metabolic functions and cell cycle arrest leading to apoptotic and necrotic cell death. Our observations give incentive to further studies addressing the efficacy of cis-UCA in intravesical treatment of bladder carcinoma.

## Methods

### Cell culture and treatments

5637 bladder carcinoma cell were cultured as monolayers in logarithmic growth phase in Dulbecco's Modified Eagle medium (Invitrogen, Paisley, UK) supplemented with 10% fetal bovine serum (Invitrogen) and penicillin-streptomycin (Sigma-Aldrich, St. Louis, MO, USA) in a humidified incubator at 37°C and 5% CO_2_. The medium was adjusted to pH 6.5 or 7.4 with NaOH after the addition of cis-UCA (BioCis Pharma, Turku, Finland) [3] or PIPES (Sigma-Aldrich) as osmotic control. For inhibition of the JNK and ERK kinase pathways, the cells were incubated with the JNK inhibitor SP600125 (10 μM; Sigma-Aldrich) or the MAPKK inhibitor PD98059 (40 μM; Sigma-Aldrich) for 40 min before cis-UCA treatment. In the protein phosphatase activity measurements, calyculin A (Calbiochem/Merck, Darmstadt, Germany) was used at 20 nM and sodium orthovanadate (New England Biolabs) at 1 mM for 2 h during the cis-UCA pulse to inhibit phosphatases.

### Measurement of intracellular pH

Two million cells were incubated in 1 ml of Hank's balanced salt solution (HBSS; Sigma-Aldrich), pH 7.4, containing 0.35 μM 2',7'-bis-(2-carboxyethyl)-5-(and-6)-carboxyfluorescein (BCECF, acetoxymethyl ester; Molecular Probes) at room temperature for 30 min, washed twice in HBSS, and resuspended in 0.5 ml of physiological saline. Various concentrations of cis-UCA were added to HBSS buffer, the solutions were adjusted to pH 6.5 or 7.4 and 300 μl of each solution was mixed with 15 μl of the BCECF-labelled cell suspension. The cells were analysed by flow cytometry (FACScan, BD Pharmingen) within 1 h. Fluorescence intensities were calibrated by analysing BCECF-labelled cells in 50 mM potassium phosphate buffers, pH 6.2-7.8, containing 43 mM KCl and 10 μM nigericin (Molecular Probes).

### Detection of cell death

After the 2-h cis-UCA pulse in indicated concentrations and a subsequent recovery time of 20 h without cis-UCA, both the floating and attached cells were collected by trypsinization. Caspase-3 activation was detected according to manufacturer's protocol (BD Pharmingen). Briefly, the cells were washed, fixed, permeabilized and labelled with phycoerythrin-conjugated anti-active caspase-3 antibody. After labelling, the cells were washed and analysed by flow cytometry (FacsCalibur, BD Pharmingen). Mitochondrial membrane potential was measured by sequestering of tetramethyl rhodamine methyl ester (TMRM) to cells. The cells were incubated in 50 nM TMRM (Invitrogen) in PBS for 15 min at 37°C, washed and analysed by flow cytometry. The TMRM-negative population was gated as cells with lost mitochondrial membrane potential. Formation of cytoplasmic and released histone-associated DNA fragments was assayed in triplicate cell lysates and cell culture supernatants, respectively, with a photometric enzyme immunoassay (Cell Death Detection ELISA^PLUS^, Roche, Mannheim, Germany) according to the manufacturer's instructions. Background absorbance of the reaction buffers at the corresponding pH without cells and the absorbance values of each well at a non-specific reference wavelength were subtracted. For detection of necrotic cell death, the cells were placed on ice and incubated with 50 μg/ml propidium iodide (PI; Sigma-Aldrich) in PBS for 10 min. The samples were analysed directly by flow cytometry for PI labelling indicative of cellular permeabilization. Nuclear morphology of the cells was investigated by labelling with DAPI. The cells were fixed with 3% paraformaldehyde for 15 min at room temperature and resuspended in PBS. Cytospin preparates were made and the samples were mounted with DAPI Vectashield (Vector Laboratories). The samples were analysed with Zeiss LSM confocal microscope (405 nm excitation, 420 nm long-pass emission filtering, 40× oil immersion objective).

### Detection of cell survival and metabolic activity

The survival of cells was quantified as the number of viable cells by using a colorimetric method (CellTiter 96 AQueous One Solution Cell Proliferation Assay; Promega) that quantifies the formation of a formazan product from 3-(4,5-dimethylthiazol-2-yl)-5-(3-carboxymethoxyphenyl)-2-(4-sulfophenyl)-2H-tetrazolium (MTS) in live cells. The absorbance at 490 nm was read in flat-bottom 96-well plates at 4 h after the addition of the assay reagent. Background absorbance (medium without cells) was subtracted from the measurements.

For measurement of metabolic activity, the MTS assay was done in triplicate samples on 24-well plates. After a 2-h cis-UCA pulse at pH 6.5, the cell medium was changed and the assay reagent was added. The absorbance was measured hourly at 490 nm up to 4 h of incubation at 37°C. Background absorbance (medium without cells) was subtracted from the measurements.

### Cell cycle analysis

For cell cycle analysis, the cells were synchronized by serum starvation for 48 h, treated with cis-UCA for 2 h and let to recover for the next 24 h. After collection, the cells were disrupted and the nuclei labelled for DNA content with PI by resuspension to sodium citrate buffer (40 mM Na-citrate, 0.3% Triton X-100, 50 μg/ml PI). After incubation at room temperature for 10 min, the samples were immediately analysed by flow cytometry.

### Western blotting

Whole-cell lysates were prepared by lysing the cells in Laemmli sample buffer and boiling for 10 minutes after which proteins were separated by SDS-PAGE. Western blotting was performed using antibodies against p44/42 ERK1/2, phosphorylated Thr202/Tyr204 ERK1/2, p46/54 JNK1/2, phosphorylated Thr183/Tyr185 JNK1/2 (all from Cell Signaling Technologies), and actin (Sigma-Aldrich). Secondary antibodies were purchased from Promega.

### Measurement of phosphatase activity

Analysis of serine/threonine phosphatase 2A and tyrosine phosphatase activity was conducted with assay kits (product no. V2460 and V2471, respectively; Promega) according to manufacturer's protocol. In this assay, cell lysate is added on artificial phosphorylated substrates, and the generation of free phosphate is determined by measuring the absorbance of a complex formed by phosphate, molybdate, and malachite green [10-12].

### Statistical analysis

The statistical significance of differences in the data was calculated with a two-way Student's t-test, with p < 0.05 considered to indicate significant differences.

## Results

### cis-UCA induces intracellular acidification in 5637 bladder carcinoma cells

**Figure 1:**
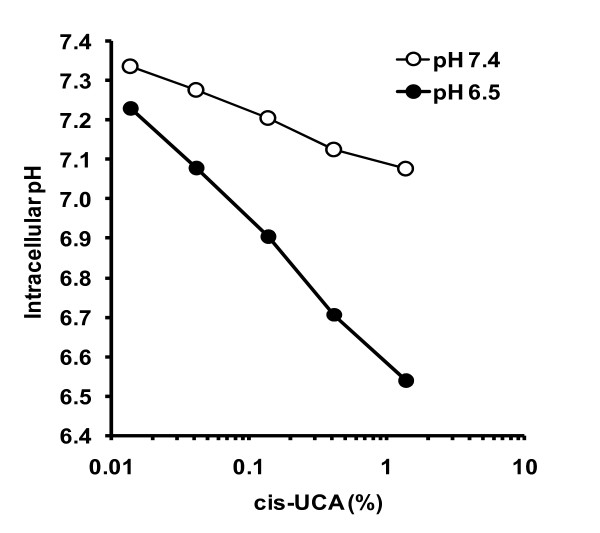
**cis-UCA induces intracellular acidification in 5637 bladder carcinoma cells**. 5637 cells were labelled with BCECF prior to treatment with cis-UCA at indicated concentrations in buffer solutions adjusted to pH 7.4 or pH 6.5. After one hour the samples were analysed by flow cytometry for intracellular pH.

According to the protodynamic concept, the prerequisite of the anticancer action of a cell-permeable weak acid such as cis-UCA is the induction of intracellular acidification. We have recently reported that cis-UCA impairs the survival of 5637 bladder cancer cells at pH 6.5 but not at pH 7.4 *in vitro *[3]. To demonstrate that cis-UCA is also able to decrease the intracellular pH, the 5637 cells were labelled with a pH sensitive fluorescent probe BCECF and treated with various concentrations of cis-UCA. In these experiments, dose-dependent acidification by up to around 0.8 pH units was seen within 1 h after the addition of cis-UCA at pH 6.5 but only by less than 0.2 units at pH 7.4 (Figure 1), confirming the pH-lowering effect of cis-UCA in bladder cancer cells. All subsequent experiments in this study were performed at pH 6.5 unless otherwise indicated.

### cis-UCA induces both apoptotic and necrotic cell death in 5637 bladder carcinoma cells

**Figure 2:**
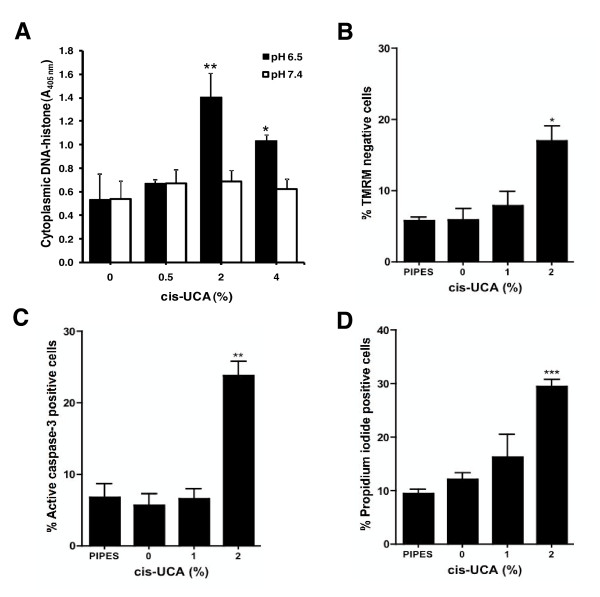
**cis-UCA induces both apoptotic and necrotic cell death in 5637 bladder carcinoma cells**. The cells were treated with indicated concentrations of cis-UCA in buffer solutions adjusted to pH 7.4 or pH 6.5 for 2 h and let to recover in the absence of cis-UCA for 20 h. The cell culture supernatants and cell lysates were analysed for formation of cytoplasmic histone-associated DNA fragments with a photometric enzyme-immunoassay **(A.)**. Alternatively, the cells treated with cis-UCA or PIPES (corresponding to 2% cis-UCA equimolar concentration) at pH 6.5 were collected and labelled with TMRM for mitochondrial membrane potential **(B.)**, analysed for caspase-3 activation **(C.) **or labelled with PI for cell permeabilization as a measure of non-apoptotic cell death **(D.)**. The samples were analysed by flow cytometry (Mean ± SEM; n = 3; Student's t-test * p < 0.05, ** p < 0.01, *** p < 0.001). The pictures are representative of two independent experiments.

Next, we examined the mechanisms of cell death in cis-UCA-treated 5637 cells. A 2-h pulse treatment model, which we have previously established [3], was used in these studies. Briefly, 5637 cells were first incubated for 2 h in the presence of 0-4% cis-UCA, followed by a 20-h recovery without cis-UCA. We were able to detect formation of cytoplasmic DNA-histone complexes indicative of apoptotic cell death after 2% cis-UCA treatment at pH 6.5, but not at pH 7.4 (Figure 2A). In the mildly acidic extracellular pH 6.5, also mitochondrial depolarization (Figure 2B) and activation of the caspase-3 (Figure 2C) were detected in cis-UCA-treated cells. These events are typical of apoptotic cell death. However, cell permeabilization indicated by propidium iodide labelling of the cells occurred with 2% cis-UCA, suggesting that cis-UCA also induces necrotic cell death (Figure 2D). This result was supported by the observation that the amount of cell-released DNA-histone complexes, measured in the extracellular medium, increased with 2% and 4% cis-UCA (data not shown). To eliminate the possibility that these effects were caused by increased extracellular osmolality due to high concentrations of cis-UCA, cells were treated with an equimolar concentration of PIPES, which is otherwise nontoxic to cells. In these experiments, PIPES affected neither cell viability nor cellular markers of apoptosis and necrosis (Figure 2).

To further analyse the biochemical events that take place after 2% cis-UCA treatment, the nuclei were labelled with DAPI and imaged by confocal microscopy (Additional file 1: Figure S1). The results showed that a large fraction of the cis-UCA-exposed 5637 cells displayed nuclei with characteristic apoptotic features, including nuclear condensation and fragmentation. To investigate caspase signalling upstream of caspase-3, the activation-induced cleavage of caspase-8 was monitored by Western blotting. Interestingly, we were unable to detect caspase-8 cleavage, indicating that caspase-8 is not activated in response to cis-UCA (data not shown). This suggests that the extrinsic apoptotic pathway is probably not involved in cis-UCA-induced cell death. A modest cleavage of poly(ADP-ribose) polymerase-1 (PARP1) after treatment with 2% cis-UCA was detected (data not shown), suggesting that in our experimental setup, the cleavage of PARP1 is more likely to indicate general cellular stress than progression of classical apoptotic cell death.

### 5637 bladder carcinoma cells enter cell cycle arrest in response to cis-UCA

**Figure 3:**
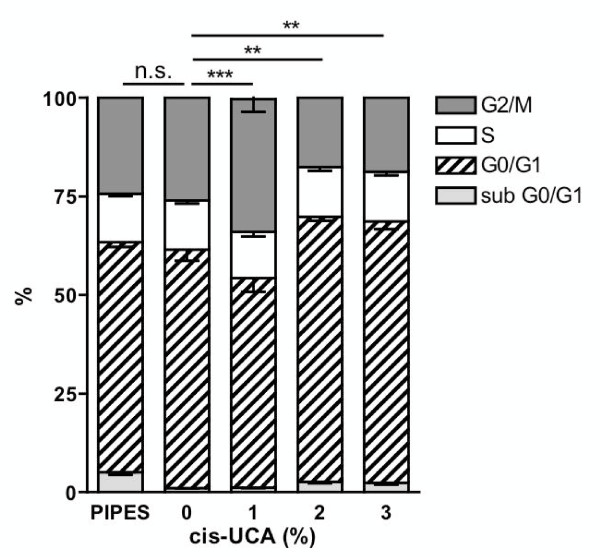
**Cell cycle progression of 5637 bladder carcinoma cells is inhibited in response to cis-UCA**. The cells were synchronized, treated with 0-3% cis-UCA or PIPES (corresponding to 3% cis-UCA equimolar concentration) for 2 h, and let to recover in the absence of treatments for 24 h. After incubation the collected cells were disrupted in sodium citrate buffer and the PI labelled nuclei were analysed by flow cytometry for DNA content. Cell cycle phases were gated and the percentage of cells in each phase quantified. Statistical analysis of the percentage of nuclei in G2/M cell cycle phase is presented (Mean ± SEM; n = 6; Student's t-test n.s. non-significant, ** p < 0.01, *** p < 0.001).

As cis-UCA was able to trigger cell death, we sought to determine whether cell cycle progression of the 5637 cells was also affected by cis-UCA. For this purpose, the cells were synchronized to G0/G1 by serum starvation, and the nuclei were analysed after the 2-h cis-UCA pulse followed by 24-h recovery for DNA content by PI labelling and flow cytometry. Interestingly, already 1% cis-UCA induced G2/M block, as the cells accumulated in this phase (Figure 3). Higher concentrations of cis-UCA slightly increased the fraction of cells in the G0/G1 phase, potentially indicating a block at the cell cycle entry after synchronization. In addition, the higher concentrations are also likely to induce a general cell cycle block due to short-circuits in cell cycle control. Western blotting studies showed that cis-UCA does not cause the accumulation of the cyclin-dependent kinase inhibitors p21(WAF1/Cip1) and p27(Kip1), which were highly expressed already in untreated 5637 cells (data not shown), indicating that the arrest is a consequence of general disturbances in cell control mechanisms rather than specific triggering of emergency break mechanisms, such as expression of p21 or p27.

### cis-UCA reduces metabolic activity and promotes ERK and JNK signalling in 5637 bladder carcinoma cells

**Figure 4:**
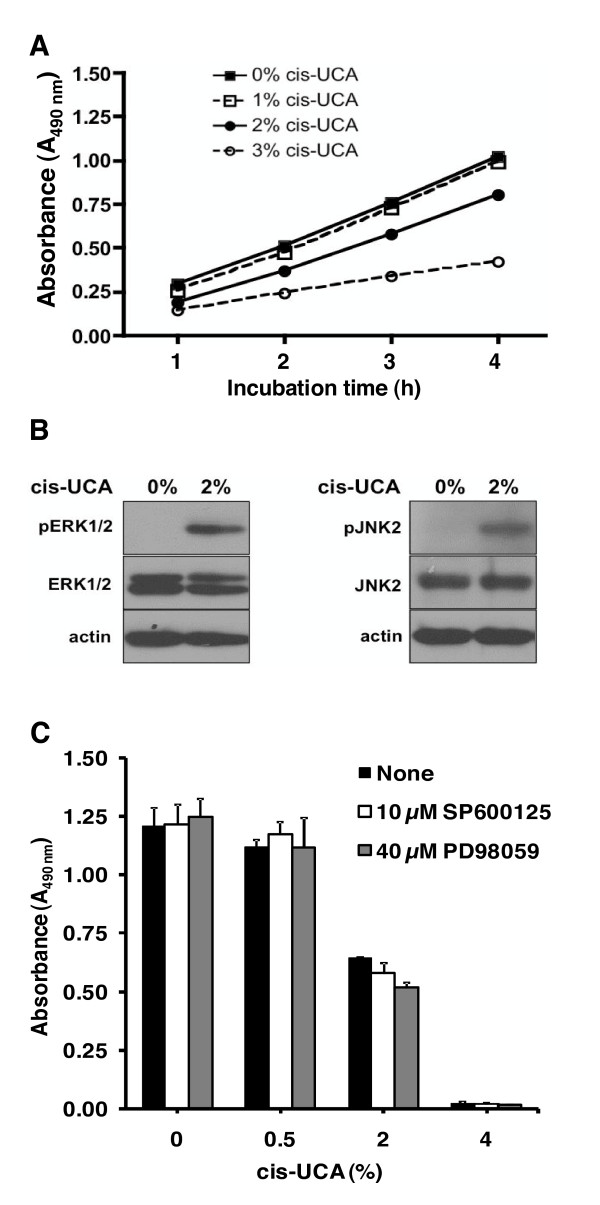
**Intracellular acidification by cis-UCA activates the ERK and JNK signalling pathways**. **(A.) **The 5637 cells were treated with 0-3% cis-UCA at pH 6.5 for 2 h after which the culture medium was changed and the MTS/PMS reagent added to cells. Absorbance (490 nm) was measured hourly up to 4 h (Mean ± SEM; n = 3, representative of two independent triplicate experiments). **(B.) **The 5637 cells were treated with 2% cis-UCA at pH 6.5. After harvesting, whole cell lysates were prepared, and ERK and JNK activity was monitored by Western blotting against phospho-ERK, ERK, phospho-JNK, and JNK. Actin expression was measured as loading control. **(C.) **The cells were treated with JNK inhibitor SP600125 or ERK inhibitor PD98059 for 40 min before addition of 2% cis-UCA at pH 6.5 for the following 2 h. Twenty hours later, cell viability was monitored by the MTS assay (Mean ± SD; n = 3).

The metabolic activity of the 5637 cells was followed for 4 h after the 2-h cis-UCA pulse using a MTS assay. A clear inhibition of the cumulative cellular reducing power was monitored by hourly measurements after 2% and 3% cis-UCA treatments (Figure 4A) without any visible effects on cell viability at these early time points. To study whether the observed metabolic inhibition was associated with activation of any major signalling pathways, we measured the effects of cis-UCA on stress and mitogen-inducible protein kinases. For this purpose, we collected whole-cell lysates immediately after the 2-h pulse with 2% cis-UCA and analysed the phosphorylation and overall expression levels of ERK1/2 and JNK1/2 by Western blotting. Interestingly, we detected a marked increase in the phosphorylated forms of ERK1/2 and JNK2 in response to cis-UCA treatment (Figure 4B), suggesting strong activation of these pathways. In contrast, the phosphorylation of JNK1 was only increased to a very low degree (data not shown), and the phosphorylation of Akt was not affected by cis-UCA (data not shown).

It is well known that the small-molecule inhibitors SP600125 and PD98059 are able to prevent the activity of JNK and ERK, respectively. To investigate if we could counteract the effect of cis-UCA on cell viability by inhibiting JNK and ERK1/2, the 5637 cells were incubated with cis-UCA in the presence of SP600125 and PD98059, after which cellular viability was measured by MTS assay. Surprisingly, the inhibitors were unable to prevent cis-UCA-mediated loss of cellular viability (Figure 4C). The inhibitors alone were non-toxic to the cells (Figure 4C and data not shown). Moreover, SP600125 and PD98059 did not affect the cis-UCA-mediated increase in phosphorylation of ERK and JNK (data not shown). Together, these data indicate that cis-UCA is not capable of directly activating these signalling pathways.

### cis-UCA inhibits cellular phosphatase activity in 5637 bladder carcinoma cells

**Figure 5:**
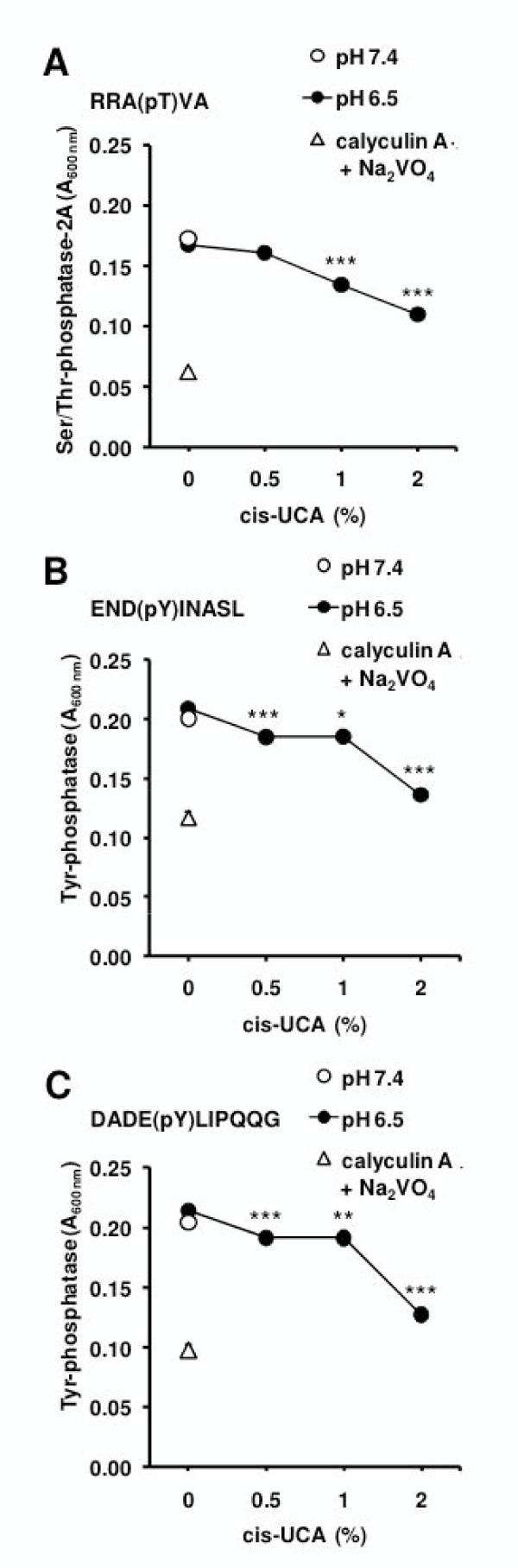
**cis-UCA inhibits phosphatase activity in 5637 bladder carcinoma cells**. To measure the effect of cis-UCA on phosphatase activity, the 5637 cells were treated with 0.5-2% cis-UCA or phosphatase inhibitors, 20 nM calyculin A and 1 mM orthovanadate (Na_2_VO_4_), at pH 6.5 for 2 h. The cells were subsequently lysed, incubated with the substrate peptide for Ser/Thr phosphatase 2A **(**RRA(pT)VA; **A.)**, or Tyr phosphatases **(**END(pY)INASL; **B**. and DADE(pY)LIPQQG; **C.) **for 30 min, and the absorbance was measured 20 min after the end of the substrate reaction (Mean ± SD; n = 3; Student's t-test * p < 0.05, ** p < 0.01, *** p < 0.001). Ser/Thr phosphatase 2A activity (A.) was measured in a reaction buffer without MgCl_2 _to inhibit phosphatase 2C activity.

Thus far we have demonstrated that the action of cis-UCA in 5637 cells is associated with increased phosphorylation of ERK1/2 and JNK2, but the inhibition of these kinase pathways did not prevent cis-UCA-induced decrease in cell survival (Figure 4C). To provide an explanation for these unexpected data, we hypothesized that the cis-UCA-induced intracellular acidification would negatively regulate the phosphatase activity in cancer cells, which would then lead to enhanced protein phosphorylation of ERK1/2 and JNK2. To investigate this possibility, we analysed the activity of protein tyrosine phosphatases as well as serine/threonine phosphatase 2A in 5637 cell lysates prepared immediately after the 2-h cis-UCA pulse. We detected a significant decrease in serine/threonine phosphatase 2A activity with 1-2% cis-UCA as well as a decreased activity towards the two tested tyrosine phosphatase substrate peptides with 0.5-2% cis-UCA (Figure 5A-C). These results suggest that cis-UCA indeed inhibits serine/threonine and protein tyrosine phosphatase activity. It is therefore likely that the reduced phosphatase activity indirectly promotes the major MAPK signalling path-ways.

## Discussion

In the present study, we aimed at characterizing the cell signalling processes that contribute to the previously reported cytotoxic effects of cis-UCA in cultured bladder cancer cells [3]. The 2-h pulse treatment model in the moderately differentiated human transitional bladder cancer cells was chosen to mimic conventional clinical intravesical instillation chemotherapy treatment of patients with non-muscle-invasive bladder cancer. We found that the protodynamic use of cis-UCA initiates intracellular acidification in bladder carcinoma cells, followed by modulation of several key processes including cell cycle, metabolic activity, and cell death. A 2-h pulse with 2% cis-UCA approximately triples the amount of apoptosis of the control level, but also promotes non-apoptotic cell death. The continuum between cell death responses can be both a consequence of secondary necrosis due lost selective membrane permeability in apoptotic cells (upon prolonged incubation), as well as the triggering of unspecific cell death within the cell population. When we analysed caspase activity and other markers of apoptosis, we found that cis-UCA induces the activation of caspase-3, causes mitochondrial depolarization and formation of cytoplasmic DNA-histone complexes, while caspase-8 activation is not affected. In addition, 2-3% cis-UCA treatment results in PARP1 cleavage. These data suggest that cis-UCA-mediated apoptosis occurs via the intrinsic apoptotic pathway, concurring with the idea of the mitochondrial pathway as the major mediator of stress-induced apoptosis.

Importantly, cis-UCA not only caused cell death, but also had cytostatic effects. We found that 2-3% cis-UCA severely impaired the metabolic activity of the 5637 bladder carcinoma cells. This effect could be detected already 1 h after the cis-UCA pulse, and the relative difference compared to the untreated cells grew over time. The induction of both apoptotic and partly necrotic responses in the bladder carcinoma cells reflect the effects of cis-UCA treatment on cellular metabolism, as mitochondrial functions play a central role in triggering of both apoptotic and necrotic cell death. Moreover, cell cycle progression was disturbed by cis-UCA, but it did not seem to be mediated by p53-dependent mechanisms, as we found that the compound had no effect on the cyclin-dependent kinase inhibitors p27 and p21, both of which are important mediators of p53-dependent cell cycle arrest. However, as the untreated 5637 bladder carcinoma cells expressed high levels of the p27 and p21 in the absence of cell cycle arrest, these inhibitory pathways may be impaired in this cancer cell type.

In addition to clarifying which cellular processes are targeted by cis-UCA, we were also interested in analysing the signalling pathways contributing to these effects. We discovered that the 5637 bladder carcinoma cells show very low endogenous activities of ERK, JNK, and Akt. Interestingly, cis-UCA remarkably increased the activity of both ERK and JNK, whereas the levels of phosphorylated Akt remained unchanged. This suggests that ERK and JNK pathways may mediate some of the cytotoxic effects of cis-UCA. However, inhibitors of the ERK and JNK pathways failed to block the effect of cis-UCA on cell viability. This indicated that cis-UCA might not promote ERK and JNK by activating their upstream regulators, but indirectly by inhibiting the phosphatases that inactivate these kinases. Remarkably, our measurements showed that the activity of protein tyrosine phosphatases and type 2A serine/threonine phosphatases was significantly decreased by 1-2% cis-UCA. Therefore, inhibition of phosphatase activity represents a novel mechanism for cis-UCA-mediated, intracellular acidification-dependent cytotoxic effects.

The idea of using a phosphatase-inhibiting agent as a means to kill cancer cells is relatively uncommon, as synthetic phosphatase inhibitors are known for their high, unspecific toxicity and tumour-promoting potential [13]. Nevertheless, natural products such as the protein phosphatase 2A inhibitor cantharidin have been shown to display anti-tumour effects [14]. Mutations of the H-Ras proto-oncogene increase phosphatase 2A activity and sensitize cancer cells to okadaic acid-induced apoptosis [15], the effects of cis-UCA might not be linked to these mutations, as the H-Ras mutation seems to be rather rare in bladder cancer [16]. However, transitional cell carcinoma of human urinary bladder has been reported to harbour H-Ras mutations [17], leaving the potential role of H-Ras in this context open.

In this study, we have characterized the molecular mechanisms underlying the anticancer effects of cis-UCA. In addition to causing apoptotic and necrotic cell death, cis-UCA also interferes with the metabolic activity and cell cycle progression of cancer cells. It is evident that the protodynamic action of cis-UCA has no selectivity against tumour cells *versus *normal cells. Instead, the potential of cis-UCA as an anticancer drug is based on the fact that solid tumour cells typically form a transmembrane pH gradient by acidifying the extracellular microenvironment, usually to around pH 6.7, while maintaining normal, slightly alkaline intracellular pH [18]. In these conditions, the tumour can be killed by intracellular acidification-induced injury caused by a protodynamic agent transporting hydrogen ions into the cytosol in a pH-sensitive manner. In the normal tissues, including the blood, the cytotoxicity of cis-UCA would be "neutralized", because no hydrogen ion transportation would occur in the absence of the transmembrane pH gradient.

Extensive preclinical evidence has confirmed that cis-UCA is neither mutagenic nor locally irritating. A single intravesical dose of up to high concentrations of 6% (60 mg/ml; 19) and 12% (120 mg/ml; Laihia *et al*. unpublished) cis-UCA instilled in the rat urinary bladder for 1 or 2 h, respectively, is well tolerated with no signs of clinical abnormality or macroscopic bladder damage. In addition, there is no apparent local or systemic toxicity in dogs treated with 2-12% cis-UCA intravesically twice a week for 1 h per instillation for 4 weeks (Laihia *et al*. unpublished). In light of the insignificant toxicity of cis-UCA, our cellular data, demonstrating marked cytotoxicity in bladder carcinoma cells, suggest that protodynamic therapy with cis-UCA could be a feasible intravesical treatment for bladder cancer. To conclude, our results elucidate how cis-UCA modulates numerous key cellular processes, thereby compromising the viability of bladder carcinoma cells. The detailed mechanisms behind phosphatase inactivation and mitochondrial permeabilization by cis-UCA treatment will be topics of our future studies.

## Conclusions

In conclusion, our study describes the cytotoxic and cytostatic effects of cis-UCA in bladder carcinoma cells, and elucidates the underlying molecular mechanisms. The protodynamic action of cis-UCA leads to decreased intracellular pH, impaired metabolic activity and increased in cell death. Importantly, cis-UCA inhibits the activity of serine/threonine and tyrosine phosphatases, thereby indirectly promoting ERK and JNK activity. In the future, the protodynamic action of cis-UCA could be utilized in intravesical therapy to compromise the viability of bladder carcinoma cells.

## Abbreviations

cis-UCA: cis-urocanic acid; ERK: extracellular signal-regulated kinase; JNK: c-Jun N-terminal kinase; MAPK: mitogen-activated protein kinase; PARP1: poly(ADP-ribose) polymerase-1.

## Competing interests

Jarmo Laihia and Lasse Leino are employees, board members, stock holders and patent inventors for BioCis Pharma Ltd. This study has been partially funded by BioCis Pharma Ltd.

## Authors' contributions

EP, AK, JL, LL and JE planned the study. EP, AK and JL carried out the experimental work, and EP, AK, JL, LL and JE analyzed the results. EP, AK and JL wrote the manuscript, and LL and JE edited the manuscript. All authors read and approved the final manuscript.

## Pre-publication history

The pre-publication history for this paper can be accessed here:

http://www.biomedcentral.com/1471-2407/10/521/prepub

## Supplementary Material

Additional file 1 **Figure S1: Apoptotic nuclear morphology following 2% cis-UCA treatment**. The control cells and the cells treated with 2% cis-UCA (pH 6.5) were fixed, labelled with DAPI and analysed by confocal microscopy for nuclear morphology (40× objective). The pictures are representative of two independent experiments. Click here for file

## References

[B1] De Fabo EC Noonan FP Mechanism of immune suppression by ultraviolet irradiation in vivo. I. evidence for the existence of a unique photoreceptor in skin and its role in photoimmunology J Exp Med 1983 158 84 98 10.1084/jem.158.1.84 6223114 PMC2187071

[B2] Norval M El-Ghorr AA Studies to determine the immunomodulating effects of cis-urocanic acid Methods 2002 28 63 70 10.1016/S1046-2023(02)00210-4 12231189

[B3] Laihia JK Pylkkänen L Laato M Boström PJ Leino L Protodynamic therapy for bladder cancer *In vitro *results of a novel treatment concept BJU Int 2009 104 1233 1238 10.1111/j.1464-410X.2009.08611.x 19466948

[B4] Laihia JK Kallio J Taimen P Kujari H Kähäri V-M Leino L Protodynamic intracellular acidification by cis-urocanic acid promotes apoptosis of melanoma cells *in vitro *and *in vivo* J Invest Dermatoladvance online publication, 3 June 2010 10.1038/jid.2010.151 20520626

[B5] Thangaraju M Sharma K Liu D Shen SH Srikant CB Interdependent regulation of intracellular acidification and SHP-1 in apoptosis Cancer Res 1999 59 1649 1654 10197642

[B6] Cosentini E Haberl I Pertschy P Teleky B Mallinger R Baumgartner G Wenzl E Hamilton G The differentiation inducers phenylacetate and phenylbutyrate modulate camptothecin sensitivity in colon carcinoma cells in vitro by intracellular acidification Int J Oncol 2001 19 1069 1074 11605011 10.3892/ijo.19.5.1069

[B7] Gottlieb RA Nordberg J Skowronski E Babior BM Apoptosis induced in Jurkat cells by several agents is preceded by intracellular acidification Proc Natl Acad Sci USA 1996 93 654 658 10.1073/pnas.93.2.654 8570610 PMC40107

[B8] Matsuyama S Llopis J Deveraux QL Tsien RY Reed JC Changes in intramitochondrial and cytosolic pH: early events that modulate caspase activation during apoptosis Nat Cell Biol 2000 2 318 325 10.1038/35014006 10854321

[B9] Juusola P Minkkinen P Leino L Laihia JK Determination of the dissociation constants of urocanic acid isomers in aqueous solutions Monatsh Chem 2007 138 951 965 10.1007/s00706-007-0687-1

[B10] Donella-Deana A MacGowan CH Cohen P Marchiori F Meyer HE Pinna LA An investigation of the substrate specificity of protein phosphatase 2C using synthetic peptide substrates; comparison with protein phosphatase 2A Biochim Biophys Acta 1990 1051 199 202 10.1016/0167-4889(90)90194-I 2155667

[B11] Daum G Solca F Diltz CD Zhao Z Cool DE Fischer EH A general peptide substrate for protein tyrosine phosphatases Anal Biochem 1993 211 50 54 10.1006/abio.1993.1231 8323038

[B12] Zhang ZY Thieme-Selfer AM Maclean D McNamara DJ Dobrusin EM Sawyer TK Dixon JE Substrate specificity of the protein tyrosine phosphatases Proc Natl Acad Sci USA 1993 90 4446 4450 10.1073/pnas.90.10.4446 7685104 PMC46528

[B13] Fujiki H Suganima M Carcinogenic aspects of protein phosphatase 1 and 2A inhibitors Prog Mol Subcell Biol 2009 46 221 254 full_text 19184590 10.1007/978-3-540-87895-7_8

[B14] Liu D Chen Z The effects of cantharidin and cantharidin derivates on tumour cells Anticancer Agents Med Chem 2009 9 392 396 19442040 10.2174/1871520610909040392

[B15] Rajesh D Schell K Verma AK Ras mutation, irrespective of cell type and p53 status, determines a cell's destiny to undergo apoptosis by okadaic acid, an inhibitor of protein phosphatase 1 and 2A Mol Pharmacol 1999 56 3 515 525 10462539 10.1124/mol.56.3.515

[B16] Knowles MA Williamson M Mutation of H-ras is infrequent in bladder cancer: Confirmation by single-strand conformation polymorphism analysis, designed restriction fragment length polymorphisms, and direct sequencing Cancer Res 1993 53 133 139 8093230

[B17] Boulalas I Zaravinos A Karyotis I Delakas D Spandidos DA Activation of *RAS *family genes in urothelial carcinoma J Urol 2009 181 2312 2319 10.1016/j.juro.2009.01.011 19303097

[B18] Tannock IF Rotin D Acid pH in tumors and its potential for therapeutic exploitation Cancer Res 1989 49 4373 4384 2545340

[B19] Arentsen HC Falke J Jansen CFJ Hulsbergen-van de Kaa CA Laihia JK Pylkkänen L Leino L Oosterwijk E Witjes JA Antitumour effects of cis-urocanic acid, an intravesical protodynamic drug, in experimental urothelial cell carcinoma of the bladder Eur Urol Suppl 2010 9 292 10.1016/j.juro.2011.11.080 22341270

