# Supplementary figures and images for: Molecular targets for the protodynamic action of cis-urocanic acid in human bladder carcinoma cells
Source: BMC Cancer. 2010 Oct 3;10:521. doi: 10.1186/1471-2407-10-521 (PMC2958937; doi:10.1186/1471-2407-10-521)

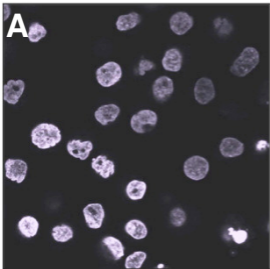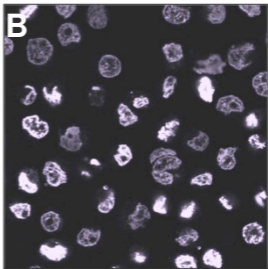

Supplement: Additional file 1 — Figure S1: Apoptotic nuclear morphology following 2% cis-UCA treatment. The control cells and the cells treated with 2% cis-UCA (pH 6.5) were fixed, labelled with DAPI and analysed by confocal microscopy for nuclear morphology (40× objective). The pictures are representative of two independent experiments. [file 1471-2407-10-521-S1.PDF]
